# Altered Processing of Auditory Distractions Under Competing Inputs in Children With Attention-Deficit/Hyperactivity Disorder

**DOI:** 10.1016/j.jaac.2025.11.003

**Published:** 2025-11-13

**Authors:** Yuanjun Kong, Xuye Yuan, Li Sun, Yiyang Wang, Jipeng Huang, Jialiang Guo, Yan Song

**Affiliations:** a State Key Laboratory of Cognitive Neuroscience and Learning, Beijing Normal University, Beijing, China; b Key Laboratory of Adolescent Cyberpsychology and Behavior ( CCNU), Ministry of Education, Wuhan, China; c Key Laboratory of Human Development and Mental Health of Hubei Province, School of Psychology, Central China Normal University, Wuhan, China; d Peking University Sixth Hospital & Peking University Institute of Mental Health, Beijing, China; e NHC Key Laboratory of Mental Health ( Peking University) & National Clinical Research Center for Mental Disorders ( Peking University Sixth Hospital), Beijing, China; f Department of Experimental Psychology, University of Oxford, Oxford, United Kingdom; g Oxford Centre for Human Brain Activity, Wellcome Centre for Integrative Neuroimaging, University of Oxford, Oxford, United Kingdom

**Keywords:** ADHD, MMN, P3a, theta activities, auditory distraction

## Abstract

**Objective:**

Children with attention-deficit/hyperactivity disorder (ADHD) have difficulties filtering irrelevant information, particularly when faced with competing inputs. These challenges may affect auditory distraction processing, especially under varying demands on visual attention. This study investigated the neural mechanisms underlying auditory distraction processing under these conditions in ADHD.

**Method:**

We recorded electroencephalography (EEG) signals from 51 children with ADHD and 48 typically developing (TD) children during the performance of a visual detection task with simultaneous irrelevant auditory stimuli under varying demands on visual attention.

**Results:**

TD children presented a significant decrease in mismatch negativity (MMN) amplitude with increasing visual attention demands. However, this effect was absent in children with ADHD, who instead exhibited an enhanced P3a response and earlier MMN and P3a latencies in response to auditory changes. Moreover, a shorter MMN latency predicted more severe inattention symptoms, whereas a larger MMN amplitude predicted more severe hyperactivity/impulsivity symptoms in children with ADHD. Time-frequency analysis revealed greater frontal theta intertrial phase coherence (ITPC) and power in response to task-irrelevant auditory changes in children with ADHD than in TD children.

**Conclusion:**

The absence of attention-demand modulation in MMN amplitude and an increased P3a response indicate atypical auditory distractibility in children with ADHD, with earlier MMN and P3a latencies suggesting excessive sensitivity to auditory distractions. Increased frontal theta ITPC and power reflect stronger neural synchronization and response to auditory distractions. Importantly, MMN amplitude and latency are linked to different aspects of ADHD symptoms. These findings provide new neurophysiological insights into altered auditory distraction processing in children with ADHD.

## Introduction

Attention-deficit/hyperactivity disorder (ADHD) is a neurodevelopmental disorder that is one of the most common neurobehavioral conditions in childhood. ADHD is characterized by atypical patterns of inattentiveness, hyperactivity and/or impulsivity ^1^. Globally, ADHD affects approximately 7.2% of children ^2^, with a prevalence of 6.3% among children in China ^3^.

Competing stimuli, such as background noise from peers during school tasks requiring sustained focus, challenge attentional control in children with ADHD. Studying in these environments requires a push-and-pull between goal-directed attention allocated to a source (e.g., schoolwork) and involuntary shifts of attention toward salient, unexpected sounds (e.g., other children’s voices). Prior studies suggest that attention difficulties in ADHD can be attributed to abnormal distractibility ^4^. Electroencephalography (EEG) has been extensively utilized to study attentional processes, and these studies have suggested that children with ADHD have abnormalities in visual goal-directed attention ^5,6^. However, the underlying brain functions involved in processing distracting auditory information in children with ADHD are not yet fully understood.

In the auditory domain, event-related potential (ERP) components, mismatch negativity (MMN) and P3a are used to assess the electrical activity of the brain that is related to change detection and involuntary orientation ^7,8^. MMN and P3a are elicited by infrequent sounds (deviant stimuli) within a repetitive sequence (standard stimuli); these components are isolated by subtracting the ERP waveform for standard stimuli from that for deviant stimuli. In adults, MMN is typically a negative deflection and has a frontal maximum amplitude distribution between 100 and 250 ms after stimulus onset. Although MMN can be elicited in individuals without conscious attention ^9,10^, numerous studies have shown that attentional modulation influences MMN in adults ^11,12^. P3a is typically a positive peak between 250 and 350 ms after the MMN waveform ^13^. In general, P3a is considered an indicator of attentional switching after change detection ^14^.

Previous studies in which MMN and P3a were used to assess auditory processing in children with ADHD often did not simulate real-life distractions. Children were typically instructed to watch a silent video or engage in similar activities that did not include a demanding task that competed with auditory input; therefore, the children did not need to allocate immediate, real-time attentional focus to visual stimuli during the presentation of auditory stimuli. Some studies reported a reduced MMN response in children with ADHD ^15,16^, whereas other studies reported no significant between-group differences ^17,18^. For P3a, Yang *et al*. ^19^ reported an attenuated response in children with ADHD during a passive auditory oddball paradigm, whereas other studies reported an increased P3a amplitude in children with ADHD during the performance of a visual task, although the auditory and visual stimuli were presented sequentially rather than concurrently ^4,20^.

Beyond ERP analyses, time-frequency analysis (TFA) can provide additional insights from single EEG trials that might be overlooked in averaged ERP analyses ^21^. Specifically, TFA allows for the calculation of frequency-specific intertrial phase coherence (ITPC) and total power to the millisecond, reflecting event-related spectral changes. Using an auditory oddball task, Fuentemilla and colleagues ^22^ reported increased theta power and phase consistency for deviant trials compared with standard trials in adults. Similarly, Hsiao and colleagues ^23^ reported that phase-locked theta oscillations are linked to detection of auditory changes. Although previous studies have suggested that attention deficits in ADHD are related to alterations in frontal theta oscillations ^24,25^, to date, no studies have examined deviant–standard differences in theta phase consistency or power in children with ADHD.

In this study, we adapted a visual detection task with simultaneous irrelevant auditory stimuli by adding manipulated visual attention demands, similar to a previous study ^26^. This cross-modal design, where auditory distractors were independent of the visual task, was chosen to better isolate distraction effects from targets ^27^. By precisely synchronizing the timing of auditory and visual stimuli, we aimed to maximize competition for attentional resources ^28^ and ensure that distractors genuinely competed for attention ^27^. This design also allowed us to compare neural responses to the same auditory stimuli under varying levels of resource competition from the visual task. We examined the EEG characteristics of auditory distraction processing in children with and without ADHD during two conditions. First, since children with ADHD often react excessively to irrelevant stimuli during tasks ^4^, we hypothesized that these children would exhibit an exaggerated brain response to auditory changes unrelated to the task compared with that exhibited by typically developing (TD) children. Second, ADHD is considered a highly context-dependent disorder of self-regulation ^29^, children with ADHD often struggle to adjust their energetic state to meet task demands ^30^, impairing their attentional abilities, especially when faced with competing stimuli ^31^. Therefore, we anticipated that, compared to TD children, children with ADHD would show less flexible modulation of neural responses to auditory distraction depending on task-related attentional demands. Third, we sought to examine the latency of MMN and P3a components to capture potential differences in the timing of neural processes, although no specific directional hypotheses were made. Finally, based on previous findings that atypical MMN amplitude and latency are associated with greater ADHD symptom severity ^31,32^, we hypothesized that if MMN amplitude or latency abnormalities were observed, they might also predict higher symptom severity in children with ADHD. To provide a more comprehensive understanding of potential neural correlates of ADHD, we also conducted exploratory analyses examining the relationship between P3a characteristics and ADHD symptom severity.

## Method

### Participants

A total of 117 children (67 with ADHD) were recruited. The study was approved by the Ethics Committees of Peking University Sixth Hospital/Institute of Mental Health and Beijing Normal University, and verbal and written informed consent was obtained from all participants and their parents. Children with ADHD were medication-naïve outpatients recruited from the clinics of Peking University Sixth Hospital/Institute of Mental Health, and TD children were recruited from primary schools in Beijing and Henan. ADHD diagnoses were confirmed by certified psychiatrists using the Kiddie Schedule for Affective Disorders and Schizophrenia for School-Age Children-Present and Lifetime Version (K-SADS-PL) ^33^, following the Diagnostic and Statistical Manual of Mental Disorders, 5th ed. (DSM-V) ^1^.These diagnoses were made as part of routine clinical evaluations and were not conducted specifically for this study. For the TD group, brief interviews with both parents and teachers were conducted to rule out any psychiatric or neurological concerns. For all participants, the severity of ADHD symptoms was quantified through parental evaluations using the ADHD Rating Scale (ADHD-RS) ^34^, and intelligence quotient (IQ) was measured with the Wechsler Intelligence Scale for Children, 4th ed. (WISC-IV) ^35^.

All participants met the following criteria: (a) normal hearing; (b) normal or corrected-to-normal vision; (c) no history of head trauma with loss of consciousness or neurological or other serious diseases; (d) no comorbidities involving schizophrenia, severe affective disorders, autism spectrum disorders, developmental disabilities, or other mental disorders; and (e) a full-scale IQ above 80. Based on these criteria, 16 children with ADHD were excluded due to comorbidities (1 with depression, 3 with anxiety disorders, 4 with autism spectrum disorder, 2 with speech developmental disabilities, 1 with learning disability, and 5 with tic disorders). Additionally, two TD children were excluded due to high ADHD symptom scores.

After further excluding 9 participants because of excessive EEG artifacts, data from 90 children (ADHD: 45, age = 10.62 ± 1.93 years, range = 7–14 years, 10 females; TD: 45, age = 10.20 ± 1.54 years, range = 7–13 years, 10 females) were analyzed. A detailed recruitment and exclusion flowchart are shown in Figure S1, available online. No significant group differences were observed in age, sex, handedness, or IQ. Children with ADHD presented significantly more severe symptoms than TD children (Table 1).

### Stimuli and procedure

**Figure 2:**
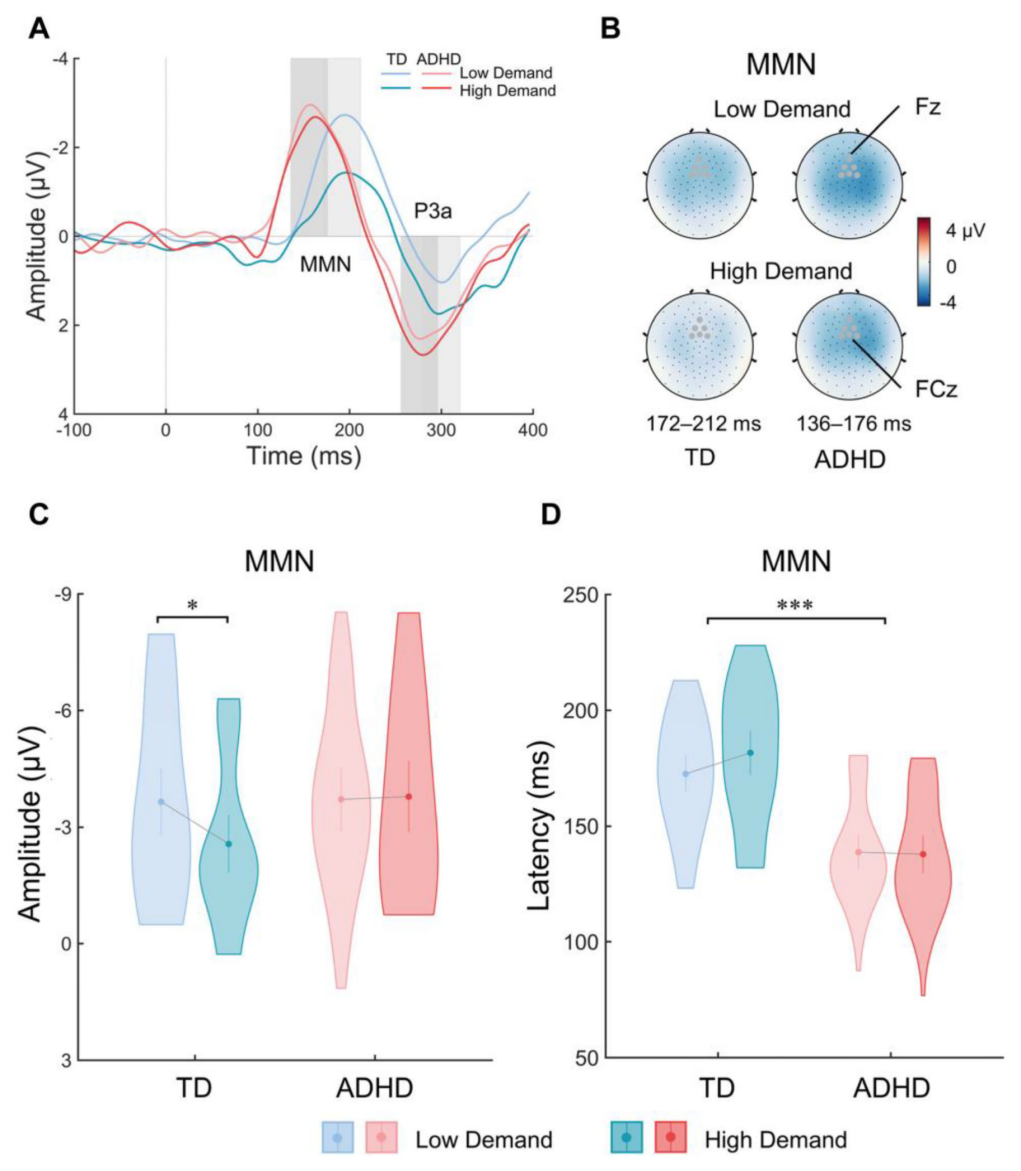
Results for the MMN Component **Note:** (A) Grand average difference waves (deviant – standard) at the frontal electrodes for the TD and ADHD groups under low- and high-demand conditions. The vertical dotted line at 0 ms indicates the time of stimulus onset. The light and dark gray rectangular areas represent the time windows used to construct topographical maps of the MMN and P3a components for both groups. (B) Topographical maps of the MMN component for the TD and ADHD groups in the low- and high-demand conditions. Grey dots indicate the frontal electrodes (Fz, 5, 12, FC1, FCz, FC2). (C) Amplitudes of the MMN component for the TD and ADHD groups in the low- and high-demand conditions. A significant interaction effect between group and visual demand on MMN amplitude was observed, revealing that TD children presented a smaller amplitude in the high-demand condition, but this effect was not observed in children with ADHD. (D) Latencies of the MMN component for the TD and ADHD groups in the low- and high-demand conditions. Compared with TD children, children with ADHD had significantly earlier MMN latencies. ADHD = attention-deficit/hyperactivity disorder; MMN = mismatch negativity; TD = typically developing. *p < .05, ***p < .001.

The participants performed a visual detection task with two conditions of varying visual attention demands, similar to a previous study (Figure 2A) ^26^. The visual stimulus was a circular search array of 12 items (1 circle, 11 diamonds) presented for 200 ms with a 600-ms interstimulus interval (ISI). A target circle appeared in 10% of the trials. In the low visual attention demand condition, the target was red; in the high-demand condition, the target was dark gray. Auditory oddball stimuli (200-ms duration, 600-ms ISI), temporally aligned with the visual stimuli, were presented simultaneously. The auditory stimuli consisted of 80% standard tones and 20% deviant tones, with frequencies counterbalanced across participants at 200 and 800 Hz. The participants were instructed to respond to the visual target while ignoring the auditory stimuli. Each visual attention condition consisted of 800 trials, including 80 target trials. The task lasted approximately 30 minutes. All participants met the minimum accuracy criterion of 60%, so none were excluded for poor task performance. See Supplement 1 for details, available online.

### EEG recording and data processing

EEG signals were recorded using a 128-channel EGI system (Electrical Geodesics, Inc.) while the participants performed the task, with Cz as the online reference and electrode impedance kept below 50 kΩ. The signals were amplified through a 0.01–400 Hz bandpass filter and digitized at 1000 Hz.

Preprocessing was performed in MATLAB (MathWorks, Inc.) using the EEGLAB toolbox ^36^. First, the 20 outermost electrodes were excluded because of their susceptibility to movement artifacts (17, 43, 48, 49, 56, 63, 68, 73, 81, 88, 94, 99, 107, 113, 119, 120, 125, 126, 127, and 128; see Figure S2, available online) ^37^. The data were subsequently downsampled to 250 Hz, re-referenced to the average of the two mastoids, and bandpass filtered (0.5–30 Hz). After manual artifact rejection and interpolation of bad electrodes, independent component analysis (ICA) was performed to identify components associated with vertical and horizontal eye movements. EEG data were then segmented from –600 to 800 ms relative to stimulus onset. An electrooculogram (EOG) was performed to further control for excessive eye movements ^5^. Then, epochs with voltages exceeding ±100 μV in any channel were excluded. To ensure that the stimuli were the same across the two conditions, only clean epochs from 90% of the no-response trials (576 standard trials and 144 deviant trials per condition) were used for ERP and time-frequency analysis. Six children with ADHD and three TD children were excluded due to more than 50% trial rejection. No group differences were found in the number of artifact-free trials per condition among the remaining participants. See Supplement 2 for details, available online.

### ERP analysis

ERP analysis was conducted in the period of –200 to 600 ms, with baseline correction applied from – 200 to 0 ms. The electrodes were selected using a grand-grand average approach ^38^. Specifically, MMN and P3a waveforms were extracted from the difference wave (deviant response – standard response), and collapsed across all groups (ADHD and TD) and conditions (low and high demand). From this collapsed average, the electrodes showing the maximal component activity were identified by ranking electrode amplitudes. This procedure reduces bias toward electrodes that maximize group differences and lowers the risk of false positives. For MMN, six frontal electrodes (Fz, 5, 12, FC1, FCz, FC2) were selected, and for P3a, six central electrodes (FCz, 7, 31, Cz, 80, 106) were selected. The electrode positions of the EGI 128-channel Geodesic Sensor Net are shown in Figure S2, and the topographical map from the collapsed average across all groups and conditions is shown in Figure S3 (both available online). We also report analyses based on a single electrode (FCz), which has been widely used in previous MMN and P3a research (see Supplement 3 for details, available online) ^39,40^. Average amplitudes within the predefined time windows (MMN: 100–250 ms; P3a: 250–350 ms) were calculated, and one-sample *t* tests were conducted to assess the presence of these amplitudes ^13,14^. To account for latency variability, MMN and P3a amplitudes in subsequent statistical analyses were calculated by averaging the values within a 20-ms window centered at the peak amplitudes for each participant, identified as the most negative (MMN) or most positive (P3a) peak within a 140-ms window centered at the group-averaged peak time ^26^. The latency was defined as the time until the difference waveform reached 50% of the individual peak amplitude ^5^. We also evaluated the split-half reliability of ERP measures to examine their internal consistency ^41^. See Supplement 4 for details, available online.

### Time-frequency analysis

Time-frequency analysis was performed for the interval from –600 to 800 ms using the FieldTrip Toolbox ^42^. The phase and power were estimated using the continuous wavelet transform. EEG data in each trial were convolved with a set of Morlet wavelets in the theta band (4–7 Hz) via 3–4.2 linearly spaced cycles with a 0.5-Hz step size and 10-ms resolution.

For the theta phase, the intertrial phase coherence (ITPC) was calculated for each time-frequency interval across trials, reflecting the consistency of phase values across trials within each electrode ^43^. To examine the difference in phase consistency between deviant and standard stimuli, the ITPC difference was obtained by subtracting the ITPC of the deviant-sound trials from that of the standard-sound trials as follows:

$$\begin{array}{c} \text{ITPC}=\left|\frac{1}{\text{N}}\times \sum_{\text{k}=1}^{\text{N}}e^{i\Phi_{k}}\right|\\ \text{ITPC}\;\text{Difference}={\text{ITPC}}_{\text{Deviant}}-{\text{ITPC}}_{\text{Standard}} \end{array}$$

where N is the number of trials and Φ_k_ is the phase angle of the signal in radians.

More positive ITPC differences indicated that phase consistency across deviant trials was greater than that across standard trials, whereas more negative values suggested greater phase consistency across standard trials than across deviant trials.

For theta power, the trial-averaged activities were removed from each trial before applying the transform to exclude phase-locked components ^44^. To estimate the effects of change-elicited auditory modulation, we calculated the theta modulation index (MI) from the sound-locked data by subtracting the power of the standard-sound trials from that of the deviant-sound trials and normalizing the result according to the average of the two types of trials. The formula is as follows:

$$\text{MI}=\frac{{\text{Power}}_{\text{Deviant}}\;-\;{\text{Power}\;}_{\text{standard}}}{\left({\text{Power}}_{\text{Deviant}}\;{\text{+}\;\text{Power}}_{\text{standard}}\right)/2}$$

More positive MIs indicated that the neural response for deviant tones was stronger than that for standard tones, whereas more negative MIs suggested stronger responses to standard tones than to deviant tones.

The ITPC difference and MI in the theta band (averaged over 4–7 Hz) were then calculated at FCz. To identify significant time clusters without prior assumptions, one-sample *t* tests and two-way repeated measures ANOVAs were performed at each time point, a method commonly used in time-frequency analyses ^45^. The one-sample *t* tests assessed whether the ITPC difference (deviant minus standard) was significantly different from zero within each group, while the two-way ANOVAs tested for main effects of group (ADHD vs. TD), attention demand (low vs. high), and their interaction. These analyses were followed by cluster-based permutation tests (1,000 iterations) controlling for multiple comparisons ^46^. Specifically, clusters of contiguous time points with significant effects (*p* < 0.05) were found, and the *t* values or *F* values within each cluster were summed to generate cluster-level statistics. During each permutation, we recalculated the cluster-based statistics to form a Monte Carlo null distribution. The *p* value (two-tailed, alpha = 0.05) was calculated as the percentile of the observed cluster-level *t* value or *F* value within the null distribution.

### Statistical analysis

Statistical analyses were performed using JASP ^47^. To compare group differences in demographics and symptom scores, independent-sample *t* tests and chi-square tests were performed. One-sample *t* tests were used to evaluate the differences between ERP components and zero. Two-way repeated measures ANOVAs were used for behavioral (d-prime, reaction time) and EEG indices (MMN/P3a amplitude, latency), with group (ADHD, TD) as a between-subject factor and level of visual attention demand (low, high) as a within-subject factor. Simple effect analysis was performed if an interaction effect was observed. Spearman’s correlation analysis was used to assess the relationships between ADHD symptom scores and MMN/P3a in the ADHD group. All significance levels were set at 0.05.

## Results

### Behavioral results

To compare the behavioral performance of the two groups under the two conditions, two-way repeated measures ANOVAs were performed on mean reaction times (RTs) for correct trials and d-prime, with group as the between-subject factor and visual attention demand as the within-subject factor. For RTs (Figure 2B), significant main effects of group (*F*_(1,88)_ = 17.045, *p* < 0.001, ${\text{η}}_{\text{p}}^{2}=0.162$) and visual attention demand (*F*_(1,88)_ = 353.981, *p* < 0.001, ${\text{η}}_{\text{p}}^{2}=0.801$) were found, suggesting that children with ADHD had slower RTs than TD children did, and RTs in the high-demand condition were significantly slower. For d-prime (Figure 2C), TD children performed better than children with ADHD did, and task performance significantly deteriorated as the visual demand increased from low to high, which was supported by significant main effects of group (*F*_(1,88)_ = 16.528, *p* < 0.001, ${\text{η}}_{\text{p}}^{2}=0.158$) and visual attention demand (*F*_(1,88)_ = 120.180, *p* < 0.001, ${\text{η}}_{\text{p}}^{2}=0.577$). No significant interactions between group and visual attention demand were found for RTs (*F*_(1,88)_ = 0.007, *p* = 0.934, ${\text{η}}_{\text{p}}^{2}=0.010$) or d-prime (*F*_(1,88)_ = 0.904, *p* = 0.344, ${\text{η}}_{\text{p}}^{2}<0.001$).

### Modulation of MMN by visual attention demands

The difference waves between ERPs evoked by auditory deviant tones and standard tones over the frontal scalp for both groups under the two conditions are illustrated in Figure 2A. To quantify the presence of the MMN component, one-sample *t* tests were performed on the difference waveforms for each group and condition. The mean difference waves within 100–250 ms were significantly different from zero in both conditions for both children with ADHD (low demand: *t*_(44)_ = –4.667, *p* < 0.001, Cohen’s *d* = –0.695; high demand: *t*_(44)_ = –2.999, *p* = 0.004, Cohen’s *d* = –0.447) and TD children (low demand: *t*_(44)_ = –4.563, *p* < 0.001, Cohen’s *d* = –0.680; high demand: *t*_(44)_ = –2.133, *p* = 0.039, Cohen’s *d* = –0.318). Topographical maps of the MMN components for both groups and conditions are shown in Figure 2B.

Two-way repeated measures ANOVAs were used to compare the differences in the amplitude and latency of MMN between the groups under the two conditions. There was a significant interaction effect between group and visual attention demand on the MMN amplitude (*F*_(1,88)_ = 4.410, *p* = 0.039, ${\text{η}}_{\text{p}}^{2}=0.048$; Figure 2C). Simple effect analysis revealed a significantly smaller MMN amplitude in the high-demand condition compared with the low-demand condition in TD children (*p* = 0.010) but not in children with ADHD (*p* = 0.853). No significant main effect of group (*F*_(1,88)_ = 1.531, *p* = 0.219, ${\text{η}}_{\text{p}}^{2}=0.017$) or demand (*F*_(1,88)_ = 3.413, *p* = 0.068, ${\text{η}}_{\text{p}}^{2}=0.037$) was observed for the MMN amplitude. Complementary analyses within the ADHD group showed no significant association between MMN amplitude and task performance, ruling out the possibility that the lack of demand-related modulation in this group was due to variability in behavioral performance (see Supplement 5, available online). Unexpectedly, there was a significant main effect of group on MMN latency (*F*_(1,88)_ = 62.991, *p* < 0.001, ${\text{η}}_{\text{p}}^{2}=0.417$; Figure 2D), indicating earlier detection of auditory changes in children with ADHD. No significant main effect of demand (*F*_(1,88)_ = 1.701, *p* = 0.196, ${\text{η}}_{\text{p}}^{2}=0.019$) or interaction between group and demand (*F*_(1,88)_ = 2.520, *p* = 0.116, ${\text{η}}_{\text{p}}^{2}=0.028$) was found for MMN latency.

These results revealed a distinct pattern in the MMN response in children with ADHD. TD children exhibited a clear modulation of MMN amplitude according to visual attention demand, with a decrease in amplitude as demand increased. However, this trade-off pattern was absent in children with ADHD, who maintained a stable MMN response, reflecting an impaired ability to allocate attentional resources in the presence of competing inputs. Moreover, the MMN latency in children with ADHD was significantly earlier than that in TD children, indicating excessive sensitivity to irrelevant auditory changes.

### Modulation of P3a by visual attention demands

**Figure 3:**
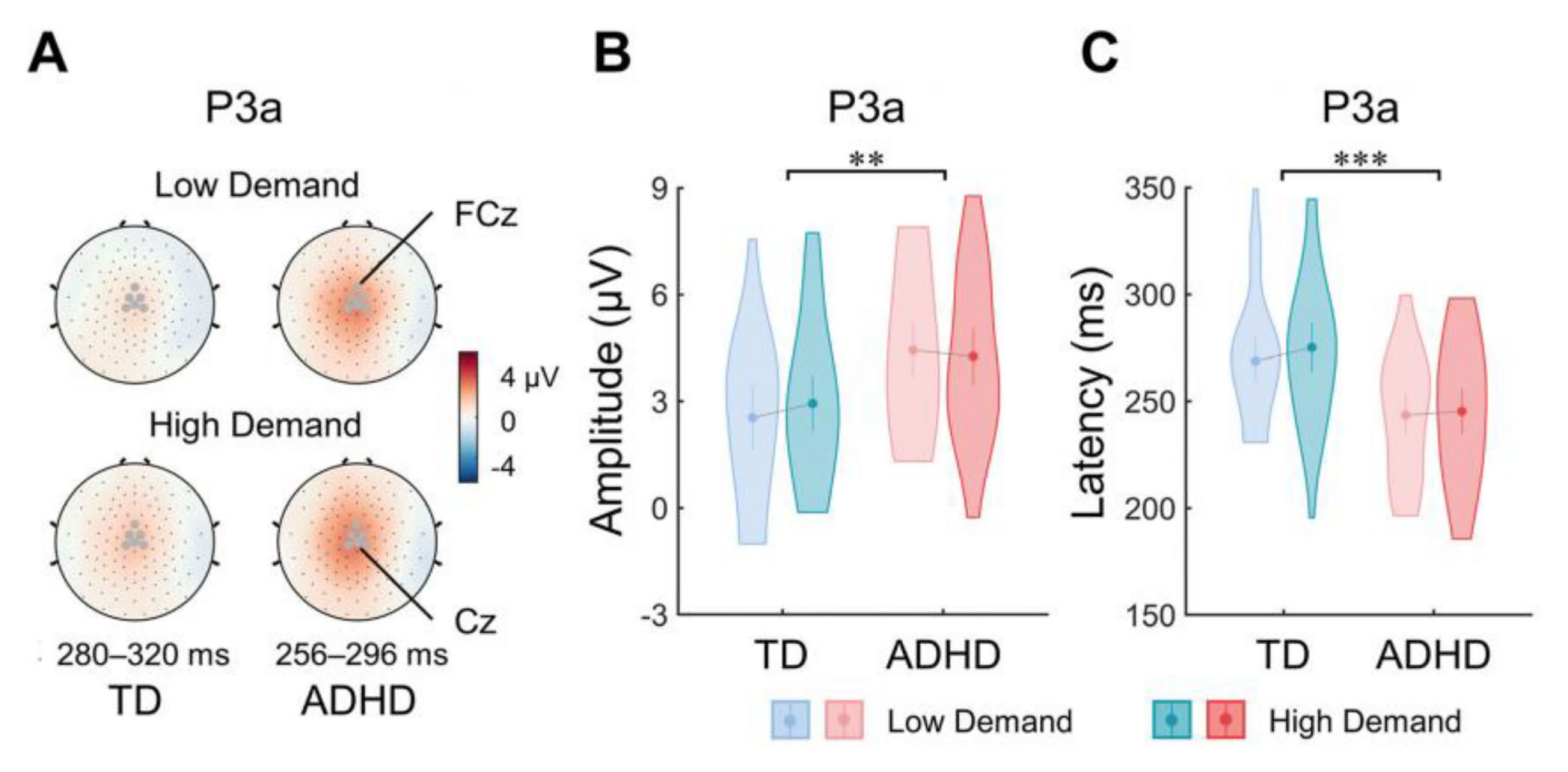
Results for the P3a Component **Note:** (A) Topographical maps of the P3a component for the TD and ADHD groups in the low- and high-demand conditions. Grey dots indicate the central electrodes (FCz, 7, 31, Cz, 80, 106). (B) Amplitudes of the P3a component for the TD and ADHD groups in the low- and high-demand conditions. Compared with TD children, children with ADHD presented a greater P3a amplitude. (C) Latencies of the P3a component for the TD and ADHD groups in the low- and high-demand conditions. Compared with TD children, children with ADHD presented earlier P3a latency. ADHD = attention-deficit/hyperactivity disorder; TD = typically developing. **p < .01, ***p < .001.

One-sample *t* tests were conducted on ERP difference waveforms to quantify the presence of the P3a component for each group in each condition. The mean difference waves within 250–350 ms were significantly different from zero for children with ADHD in both conditions (low demand: *t*_(44)_ = 5.955, *p* < 0.001, Cohen’s *d* = 0.888; high demand: *t*_(44)_ = 5.930, *p* < 0.001, Cohen’s *d* = 0.884) and for TD children in the high-demand condition (*t*_(44)_ = 4.381, *p* < 0.001, Cohen’s *d* = 0.653); the difference was marginally significant for TD children in the low-demand condition (*t*_(44)_ = 1.968, *p* = 0.055, Cohen’s *d* = 0.293). The topographical maps of the P3a components for both groups and conditions are shown in Figure 3A.

To compare P3a amplitude and latency between groups and conditions, we performed two-way ANOVAs. A larger P3a amplitude was found in children with ADHD than in TD children (*F*_(1,88)_ = 10.495, *p* = 0.002, ${\text{η}}_{\text{p}}^{2}=0.107$; Figure 3B), indicating greater auditory distractibility. There was no significant main effect of demand (*F*_(1,88)_ = 0.146, *p* = 0.703, ${\text{η}}_{\text{p}}^{2}=0.002$) or interaction effect between group and demand (*F*_(1,88)_ = 0.931, *p* = 0.337, ${\text{η}}_{\text{p}}^{2}=0.010$). A notably significant main effect of group was observed for P3a latency (*F*_(1,88)_ = 20.434, *p* < 0.001, ${\text{η}}_{\text{p}}^{2}=0.188$; Figure 3C), indicating earlier involuntary orienting of attention toward auditory distractors in children with ADHD. No significant main effect of demand (*F*_(1,88)_ = 0.894, *p* = 0.346, ${\text{η}}_{\text{p}}^{2}=0.010$) or interaction effect between group and demand (*F*_(1,88)_ = 0.287, *p* = 0.594, ${\text{η}}_{\text{p}}^{2}=0.003$) was found for P3a latency.

The complementary FCz-based analysis yielded similar results to the multi-electrode analysis (see Supplement 3, available online). The reliability estimates for MMN and P3a amplitudes and latencies were good (0.711–0.831) across groups and conditions (Table S1, available online).

### Frontal theta activity responses to distractor changes

Previous research has suggested that neural responses to auditory changes are especially pronounced within the theta band (4–7 Hz). We calculated the frontal theta ITPC difference (deviant – standard) and MI to examine group differences in theta responses to distractor changes under varying visual task demands.

**Figure 5:**
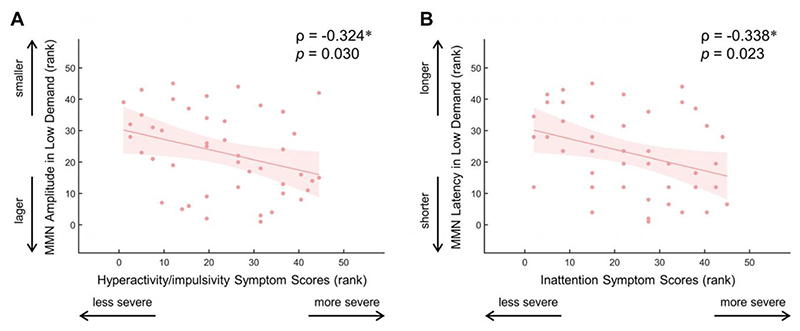
Correlation Results in Children With ADHD **Note:** (A) The larger the MMN amplitude was in the low-demand condition, the more severe the hyperactivity/impulsivity symptoms were in the ADHD group. (B) The shorter the MMN latency was in the low-demand condition, the more severe the inattention symptoms were in the ADHD group. A higher rank means a larger numerical value. The shaded areas represent 95% confidence intervals (CIs). ADHD = attention-deficit/hyperactivity disorder; MMN = mismatch negativity. *p < .05.

For the frontal theta ITPC difference, the cluster-based permutation *t test* revealed a significant positive ITPC difference for children with ADHD, while a significant negative ITPC difference for TD children (see Supplement 6, available online). Moreover, cluster-based permutation ANOVA revealed significant main effects of both group (0–270 ms, cluster *p* < 0.001; Figure 5A) and attention demand (170–300 ms, cluster *p* < 0.001; Figure 5B). Specifically, children with ADHD presented more positive ITPC differences than TD children did in both conditions. That is, relative to TD children, children with ADHD presented increased frontal phase consistency when tones changed from standard to deviant. In addition, the ITPC difference was more negative in the high-demand condition than in the low-demand condition for both groups, indicating that as the visual attention demand increased, the frontal phase consistency in auditory deviant trials decreased relative to that in auditory standard trials. There was no significant interaction effect between group and visual attention demand (*p* > 0.05).

A significant positive frontal theta MI was observed in children with ADHD, but was not found in TD children (see Supplement 6, available online). Further cluster-based permutation ANOVA confirmed this group difference (330–400 ms, cluster *p* < 0.001; Figure 5C), suggesting stronger frontal theta power for deviant tones than to standard tones in children with ADHD but not in TD children. No significant effect of attention demand or interaction between group and attention demand was observed for theta MI (*p*s > 0.05).

The results of frontal theta activity indicated that children with ADHD had stronger neural responses to irrelevant deviant tones than TD children did, as evidenced by greater theta ITPC and increased theta power for deviant tones in both conditions. Additionally, as visual attention demand increased, both groups presented reduced frontal phase consistency in response to irrelevant auditory changes.

### Correlations between the severity of ADHD symptoms and MMN

Correlation analyses of ADHD symptom scores with MMN/P3a amplitude and latency were performed for children with ADHD. Hyperactivity/impulsivity symptom scores positively correlated with MMN amplitude in the low-demand condition (ρ = –0.324, *p* = 0.030; Figure 5A), suggesting that larger MMN amplitudes correlate with more severe hyperactivity/impulsivity symptoms. On the other hand, inattention symptom scores were negatively correlated with MMN latency in the low-demand condition (ρ = –0.338, *p* = 0.023; Figure 5B), indicating that children with ADHD who had shorter MMN latency were more likely to exhibit more severe inattention symptoms. These findings align with our initial hypothesis that abnormal MMN characteristics would be associated with greater ADHD symptom severity. No other significant correlations were observed (*p*s > 0.166, uncorrected). All correlation results were additionally tested using permutation-based correction for multiple comparisons (see Supplement 7 for details, available online) ^46,48,49^, corrected *p* values are provided in Table S2, available online.

## Discussion

The deployment of attention between goal-directed processing and involuntary shifts toward distractors is particularly challenging for children with ADHD. Previous studies have not fully explored how the brain responds to auditory changes in children with ADHD, especially when auditory stimuli act as distractors under varying levels of visual attention demand. In this study, we employed a varying-demand visual detection task with irrelevant auditory stimuli to investigate the brain mechanisms underlying the processing of auditory distractions in children with ADHD. Our findings revealed abnormal auditory distractibility in children with ADHD. Compared with TD children, children with ADHD presented greater P3a responses to irrelevant auditory changes. Only TD children presented a decrease in MMN amplitude as visual attention demand increased, whereas this trade-off pattern was absent in children with ADHD. Surprisingly, both MMN latencies and P3a latencies were earlier in children with ADHD, indicating faster neural responses to irrelevant auditory changes. A shorter MMN latency in children with ADHD predicts stronger inattention symptoms, whereas a larger MMN amplitude predicts severe hyperactivity/impulsivity symptoms. Further analysis revealed that compared to TD children, children with ADHD presented stronger responses to irrelevant deviant tones, as reflected by greater frontal theta ITPC and theta power. Together, these findings suggest heightened auditory distractibility and altered neural processing dynamics in children with ADHD.

Prior research has demonstrated a trade-off between auditory change detection and visual selective attention in adults ^26,50^. In the present study, we observed a similar pattern in TD children, with a reduction in auditory MMN amplitude when visual attention demand shifted from low to high. This finding is consistent with those of adult studies ^11,28^, suggesting that TD children allocate more attentional resources to visual processing as attention demand increases, diminishing the resources available for auditory processing, which is consistent with the capacity-limited push-and-pull mechanism proposed in load theory ^51,52^. However, this adaptive trade-off between goal-directed processing and distractor processing was absent in children with ADHD, consistent with our directional hypothesis. Children with ADHD showed similar MMN amplitudes under low visual attention demands and high visual attention demands, indicating that children with ADHD may struggle to filter out irrelevant sounds, reflecting an atypical sensitivity to distraction. This finding may also reflect a potential inability of children with ADHD to allocate attentional resources effectively in the presence of competing inputs, which is consistent with the nature of ADHD as a highly context-dependent disorder of self-regulation ^29^.

We also found that children with ADHD presented greater P3a amplitudes than TD children did, which is consistent with some previous studies ^4,20^ but contradicts research reporting smaller P3a amplitudes ^19^. In Yang *et al*. ^19^, children with ADHD played a computer game without task-specific demands, during which a smaller P3a response to sounds was observed. This difference may be explained by the auditory stimuli being more distracting in other studies using the distraction paradigm ^4,20^, potentially leading to larger P3a amplitudes in children with ADHD. Another possible factor is the sensitivity of responses to varying auditory stimulus parameters across studies ^53^, which may affect ADHD and TD groups differently. In our paradigm, auditory stimuli were precisely synchronized with each visual search display, maximizing competition with the visual task for attentional resources ^26,28^. Consequently, children with ADHD presented increased P3a amplitudes in response to these more disturbing distractors, indicating an exaggerated brain response to task-irrelevant sounds.

Our finding that the abnormal timing of neural responses to auditory stimuli in children with ADHD is of particular interest. Compared with healthy controls, most studies reported longer latencies ^54,55^ or no difference ^17,31^ in the latencies of MMN and P3a components in ADHD individuals. However, several studies have reported decreased MMN latency in children with ADHD ^18,56^, albeit with small sample sizes. In this study, using a task in which auditory inputs were used as distractors with a larger sample size, we found that children with ADHD exhibited shorter latencies (approximately 40 ms) for both the MMN and P3a components. This result suggests that children with ADHD respond more quickly to auditory distractors than their TD counterparts do when visual focus is needed, potentially processing these stimuli faster. This finding supports the abnormal distractibility of children with ADHD ^4,20^, as evidenced by their excessive sensitivity to auditory distractions.

Previous studies have suggested that theta activity in the frontal area is associated with cognitive control ^57^. In our study, when the task-irrelevant sound changed from standard to deviant, children with ADHD presented greater frontal theta ITPC differences and frontal theta MI than TD children did, which extends previous findings from visual modality studies in children with ADHD ^24,25^ and provides neural evidence for abnormal theta activity in the auditory modality. As visual attention demand increased, the theta ITPC difference decreased in both groups, suggesting that children focused more on the visual task and responded less to irrelevant sounds. We suggest that the heightened theta phase consistency and power in response to irrelevant auditory changes also reflect excessive sensitivity to auditory distractions, as supported by the MMN and P3a results; taken together, these findings potentially reflect executive function deficits in children with ADHD, particularly difficulty in inhibiting inappropriate responses ^58^.

Our study revealed intriguing relationships between MMN characteristics and different ADHD symptoms. Specifically, when children with ADHD were instructed to engage in a visual attention task, stronger responses to auditory distractors (larger MMN amplitudes) were linked to more severe hyperactivity/impulsivity symptoms, whereas faster responses (shorter MMN latencies) were linked to more severe inattentive symptoms. In contrast, in a study in which auditory stimuli were not presented as distractors, Hsieh and colleagues ^31^ reported that smaller MMN amplitudes in adults with ADHD were linked to more severe inattention symptoms. Similarly, Yamamuro *et al*. ^32^ reported attenuated MMN amplitudes and prolonged latencies in the ADHD group, which correlated with more severe hyperactivity/impulsivity symptoms. These findings consistently suggest that atypical MMN characteristics are associated with more severe ADHD symptoms. Our results strengthen the notion that MMN is sensitive to ADHD symptoms and highlight new directions for research into the role of auditory stimuli in ADHD.

Although we synchronized the timing of visual arrays and irrelevant sounds to maximize the competition for attentional resources, the visual and auditory stimuli used in the current study were relatively simple. These laboratory settings may not fully capture the complexity of real-world attentional challenges, such as those faced in classrooms. Future research could benefit from incorporating more naturalistic tasks and a wider variety of stimuli to better understand how auditory distraction affects attention in everyday situations, particularly in children with ADHD. In addition, our design increased distraction and competition for attention by only manipulating visual task demands, which did not allow for direct isolation of distraction effects behaviorally. Future studies could adopt alternative designs, such as manipulating the salience or complexity of distractors, to investigate distraction mechanisms in children with ADHD. Moreover, since the current study focused only on auditory distractors, it would be valuable for future research to examine whether the observed distraction effects can be generalized to other types of distractors, thereby providing a more comprehensive understanding of distractibility in children with ADHD. Finally, we did not collect additional sociodemographic variables such as socioeconomic status, which should be considered in future research.

In summary, this study explored auditory distraction processing under competing inputs in children with ADHD, revealing exaggerated and faster brain responses to irrelevant auditory changes, as indicated by distinct neurophysiological markers, and identified associations between MMN and specific ADHD symptoms. These findings improve our understanding of how children with ADHD process distractions under competing inputs, support impairments in both inhibitory control ^58^ and context-dependent modulation of attention ^29^, and suggest that brain responses to auditory distractor changes may help identify symptom patterns and guide future intervention strategies. Our findings also indicate that children with ADHD possess certain often overlooked strengths. In specific contexts, children with ADHD may even have an advantage over TD children, as they can rapidly detect abnormalities in their environment and quickly disengage from the current task, which can be beneficial in situations requiring quick reactions.

## Supplementary Material

Supplementary Information

## Figures and Tables

**Figure 1:**
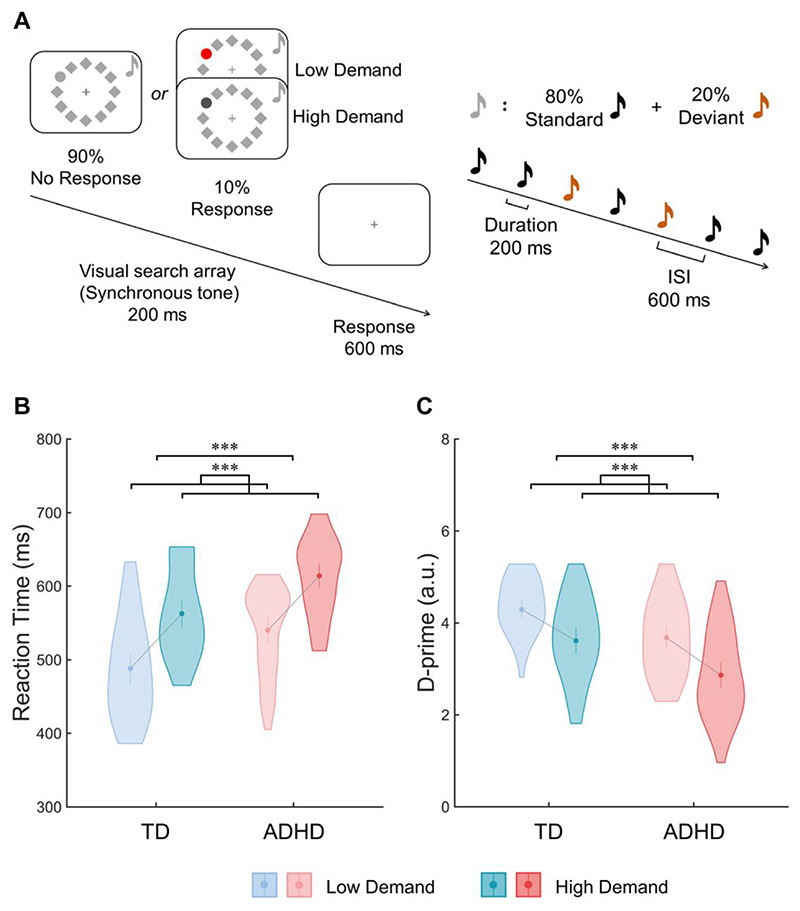
Task Paradigm and Behavioral Results **Note:** (A) Participants were instructed to focus on the visual search array and respond to the color of the target (left) and ignore the task-irrelevant auditory stimuli presented simultaneously (right). For each demand condition, the circle and diamonds remained light gray in 90% of the trials, requiring no response; in the remaining 10% of the trials, the response-target circle was red and dark gray for the low- and high-demand conditions, respectively. The auditory stimulus series consisted of 80% standard tones and 20% deviant tones, with the frequencies counterbalanced across participants at 200 and 800 Hz. Each tone was presented in sync with a visual search array for 200 ms, followed by a 600 ms interstimulus interval (ISI). (B) Reaction times for the TD and ADHD groups in the low- and high-demand conditions. Children with ADHD had significantly slower RTs than TD children did, with RTs worsening significantly as visual demand increased from low to high. (C) D-prime for the TD and ADHD groups in the low- and high-demand conditions. Task performance was significantly poorer in children with ADHD than in TD children, and performance significantly decreased as visual demand increased from low to high. The dots indicate the mean values across participants, and the error bars represent the 95% confidence intervals (CIs), which are the same in all other figures below. ADHD = attention-deficit/hyperactivity disorder; TD = typically developing. ***p < .001.

**Figure 4:**
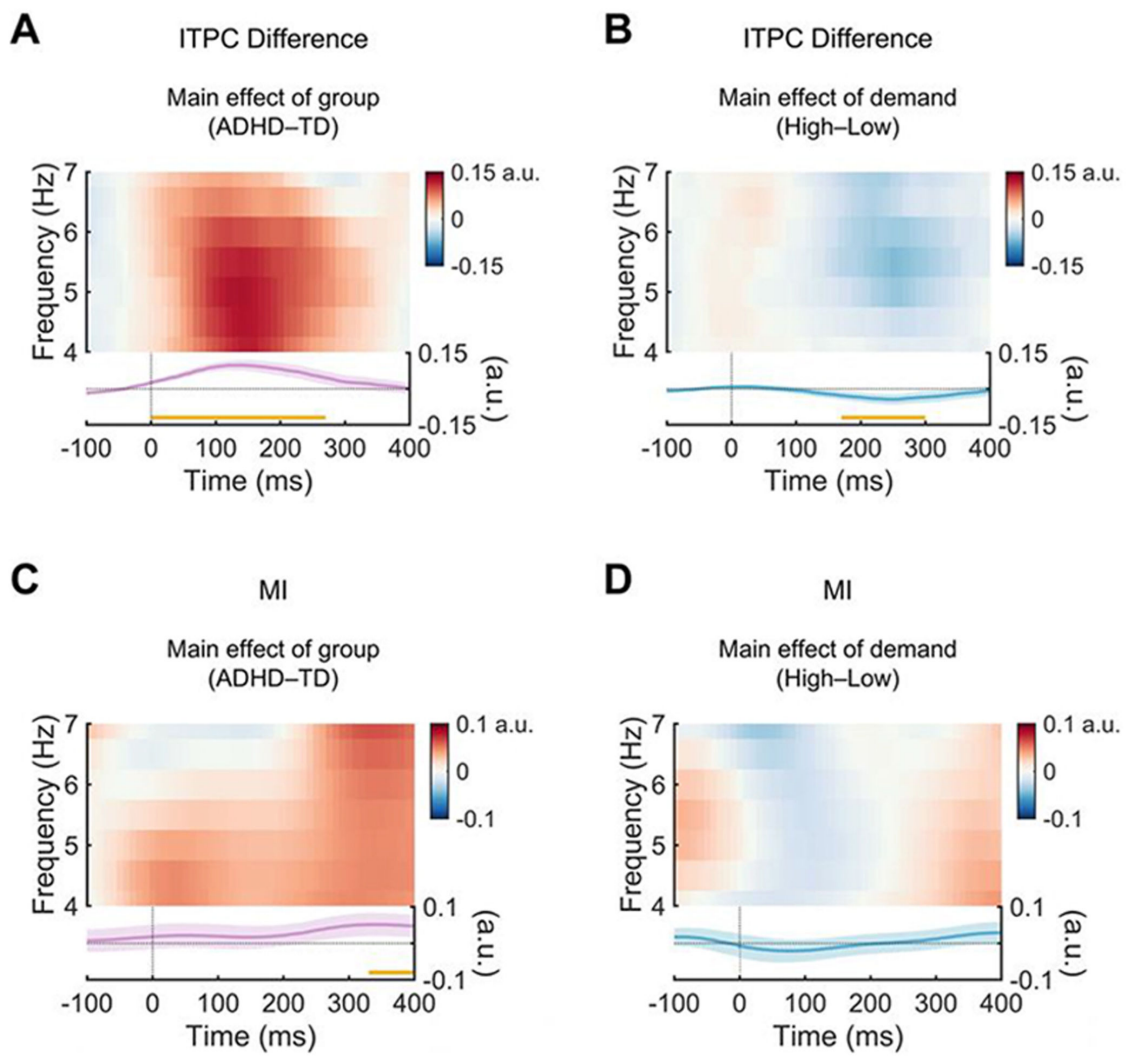
Time-Frequency Results **Note:** (A) Main effect of group on the ITPC difference, with the corresponding averaged theta-band activity shown below. Positive values indicate greater phase consistency across deviant trials in the ADHD group. Yellow lines at the bottom indicate periods with significant main effect of group (cluster *p* < .001) on the theta ITPC difference. (B) Main effect of demand on the ITPC difference, with the corresponding averaged theta-band activity shown below. Negative values in the right panels indicate greater phase consistency across deviant trials under the low-demand condition. Yellow lines at the bottom indicate periods with significant main effects of demand (cluster *p* < .001) on the theta ITPC difference. (C) Main effect of group on MI, with the time course of averaged theta activity shown below. Positive values indicate stronger neural responses to deviant tones in the ADHD group. The yellow lines at the bottom indicate the periods with significant main effect of group (cluster *p* < .001) on the theta MI. (D) Main effect of demand on MI, with the time course of averaged theta activity shown below. No significant main effect of demand on the theta MI was observed. Light-colored shading represents error bars (±1 *SE*) in all plots. ADHD = attention-deficit/hyperactivity disorder; ITPC = intertrial phase coherence; MI = modulation index; TD = typically developing.

**Table 1:** Demographic Information and Symptom Scores of the ADHD and TD Groups

Mean ± SD	ADHD *(n* = 45)	TH *(n* = 45)	Group difference (*t*/χ^2^)
Age (years)	10.62 ± 1.93	10.20 ± 1.54	1.157
Gender (male: female)	35: 10	35: 10	0.000
Handedness (left: right)	2: 43	0: 45	0.511
IQ	110.09 ± 12.41	113.27 ± 11.60	−1.255
Symptom scores			
Inattention	17.73 ± 3.09	7.00 ± 2.80	17.248***
Hyperactivity/impulsivity	11.38 ± 5.72	4.58 ± 2.83	7.149***

**Note:** Test statistics indicate the difference between the two groups. ADHD = attention-deficit/hyperactivity disorder; IQ = intelligence quotient; TD = typically developing.

*** *p* < .001.
