# Supplementary material for: Altered Processing of Auditory Distractions Under Competing Inputs in Children With Attention-Deficit/Hyperactivity Disorder
Source: J Am Acad Child Adolesc Psychiatry. Author manuscript; Available in PMC 2026 Jul 1. (PMC7619170; doi:10.1016/j.jaac.2025.11.003)
Supplement: Supplementary Information [file EMS213418-supplement-Supplementary_Information.docx]

**Altered Processing of Auditory Distractions Under Competing Inputs in Children With ADHD**

***Supplemental Materials***

**Supplement 1. Stimuli and procedure**

The participants sat 65 cm away from a 21-inch gamma linearized CRT monitor (1920 pixels × 1080 pixels, frame rate 60 Hz) in a dimly lit, noise-reduced, and electromagnetically shielded room. The participants performed a visual detection task involving two conditions that differed in the level of visual attention demand (Figure 1A). Visual attention demands were manipulated by varying the similarity between the visual target and the nontarget. In a random 90% of the trials, the visual stimuli were a circular search array shown for 200 ms with a 600-ms interstimulus interval (ISI), including a total of twelve light gray items (3.4° × 3.4°; RGB: 128, 128, 128; 13.5 cd/m^2^) presented at a visual angle of 9.2° from the fixation cross (RGB: 128, 128, 128; 13.5 cd/m^2^) on a black background (RGB: 0, 0, 0; 0.5 cd/m^2^). The twelve items consisted of one circle and eleven diamonds, with the circle randomly located at one of four positions (right visual field: 2 or 4 o’clock, left visual field: 8 or 10 o’clock) with equal probability. In the low visual attention demand condition, the target circle in the remaining 10% of the trials turned red (RGB: 237, 28, 36; 13.5 cd/m^2^), a salient color, making it easy to detect. However, in the high visual attentional demand condition, the target circle was dark gray (RGB: 60, 60, 60; 3 cd/m^2^), less distinguishable from the nontarget shapes, requiring participants to deploy more attentional resources to identify the target. The participants were instructed to maintain their gaze on the fixation cross and press a key as quickly and accurately as possible when a red or dark gray target circle appeared in the visual search array (10% of the trials). No response was required for the other 90% of the trials. The target circle was never presented in two consecutive trials.

During the visual task, passive auditory oddball stimuli were presented simultaneously with each visual search array for a duration of 200 ms with an ISI of 600 ms, ensuring that the visual and auditory stimuli were synchronized. Auditory pure tone stimuli (10 ms rise and fall times) were delivered binaurally to participants through over-ear headphones. The auditory stimuli consisted of 80% standard tones and 20% deviant tones, and their frequencies were counterbalanced across participants at 200 and 800 Hz. The order of standard and deviant tones was randomized, with the constraint that each deviant tone was preceded by at least one standard tone. Before the task began, the sound level was initially presented at a standardized volume level (70 dB SPL). Minor adjustments were made for a few participants based on comfort ^1^, given that prior evidence suggests MMN responses to frequency changes are relatively unaffected by variations in tone intensity ^2^. The participants were instructed to ignore task-irrelevant tones and focus on the visual detection task. The proportion of standard and deviant tones was the same in the target and nontarget trials. Each of the two conditions contained eight blocks of 100 trials, which were performed separately, with the order of conditions counterbalanced between participants.

**Supplement 2. EEG recording and data processing**

In this study, independent component analysis (ICA) was used to identify and remove components associated with vertical and horizontal eye movements. In addition, we also assessed an electrooculogram (EOG) to further control for excessive eye movements ^3^. Trials in which the vertical EOG (average of Fp1 and Fp2) exceeded ±70 μV during the 0–200 ms window were excluded before ICA to further control for eye blinks. After removing the vertical and horizontal eye movement ICs, epochs in which the horizontal EOG (difference between F9 and F10) exceeded ±50 μV during 200–400 ms were excluded to further control for horizontal gaze shifts.

To ensure that the stimuli were the same across the two conditions, only clean epochs from 90% of the no-response trials (576 standard trials and 144 deviant trials per condition) were used for ERP and time-frequency analysis. To ensure data quality, participants were required to have at least 288 standard and 72 deviant artifact-free trials per condition for inclusion. Six children with ADHD and three TD children were excluded due to more than 50% trial rejection. Regarding the number of acceptable trials, the ADHD group had, on average, 435 ± 68 standard and 111 ± 19 deviant trials in the low-demand condition, and 448 ± 63 standard and 116 ± 16 deviant trials in the high-demand condition. The TD group had, on average, 442 ± 49 standard and 115 ± 16 deviant trials in the low-demand condition, and 456 ± 53 standard and 118 ± 16 deviant trials in the high-demand condition. There were no significant differences between groups in the number of artifact-free trials per condition (low-demand standard: *t*_(88)_ = –0.582, *p* = 0.562, Cohen’s *d* = –0.123; low-demand deviant: *t*_(88)_ = –1.025, *p* = 0.308, Cohen’s *d* = –0.216; high-demand standard: *t*_(88)_ = –0.625, *p* = 0.534, Cohen’s *d* = –0.132; high-demand deviant: *t*_(88)_ = –0.485, *p* = 0.629, Cohen’s *d* = –0.102).

**Supplement 3.** **FCz-based analysis**

To avoid circularity, we also selected FCz as an a priori defined electrode based on previous studies (Figure S2A) ^4,5^. Topographical maps of the MMN and P3a components for both groups and conditions are shown in Figure S2B. Two-way repeated measures ANOVAs were used to compare the differences in the amplitude and latency of MMN between the groups under the two conditions. Consistent with the multi-electrode analysis, there was a significant interaction effect between group and visual attention demand on the MMN amplitude (*F*_(1,88)_ = 4.458, *p* = 0.038, η2 p = 0.048; Figure S2C). Simple effect analysis revealed a significantly smaller MMN amplitude in the high-demand condition compared with the low-demand condition in TD children (*p* = 0.012) but not in children with ADHD (*p* = 0.828). No significant main effect of group (*F*_(1,88)_ = 1.496, *p* = 0.225, η2 p = 0.017) or demand (*F*_(1,88)_ = 3.314, *p* = 0.072, η2 p = 0.036) was observed for the MMN amplitude. Importantly, there was still a significant main effect of group on MMN latency (*F*_(1,88)_ = 58.344, *p* < 0.001, η2 p = 0.399; Figure S2D), indicating the robustness of this finding. No significant main effect of demand (*F*_(1,88)_ = 2.128, *p* = 0.148, η2 p = 0.024) or interaction between group and demand (*F*_(1,88)_ = 1.252, *p* = 0.266, η2 p = 0.014) was found for MMN latency.

The topographical maps of the P3a components for both groups and conditions are shown in Figure S3A. To compare P3a amplitude and latency between groups and conditions, we conducted two-way measures ANOVAs. Consistent with the multi-electrode results, children with ADHD showed significantly larger P3a amplitudes than TD children (*F*_(1,88)_ = 7.470, *p* = 0.008, η2 p = 0.078; Figure S3B). There was no significant main effect of visual demand (*F*_(1,88)_ = 0.572, *p* = 0.452, η2 p = 0.006), nor a significant interaction between group and demand (*F*_(1,88)_ = 1.334, *p* = 0.251, η2 p = 0.015). For P3a latency, a robust main effect of group was also observed (*F*_(1,88)_ = 19.076, *p* < 0.001, η2 p = 0.178; Figure S3C). No significant main effect of demand (*F*_(1,88)_ = 2.054, *p* = 0.155, η2 p = 0.023) or interaction effect between group and demand (*F*_(1,88)_ = 0.039, *p* = 0.844, η2 p < 0.001) was found.

**Supplement 4. Reliability of ERP measures**

We calculated split-half reliability for ERP amplitudes and latencies across all conditions and groups ^6,7^. Specifically, even and odd trial averages were computed separately for standard and deviant stimuli within each visual demand condition, and difference waves (deviant minus standard) were derived for even and odd trials. The correlation between even and odd MMN/P3a amplitudes and latencies was then calculated and corrected using the Spearman-Brown formula. The reliability estimates for MMN and P3a amplitudes and latencies were good (0.711–0.831) across groups and conditions (Table S1).

**Supplement 5. Potential influence of** **task performance on MMN findings**

To assess whether task performance influenced MMN findings, we conducted two complementary analyses within the ADHD group. We stratified participants based on task performance using a median split on d-prime, comparing MMN amplitudes between individuals with relatively higher and lower performance using independent-sample *t* tests within each visual demand condition. This analysis revealed no significant difference in MMN amplitude between the subgroups (low demand: *t*_(43)_ = 1.107, *p* = 0.274, Cohen’s *d* = 0.330; high demand: *t*_(43)_ = 0.060, *p* = 0.952, Cohen’s *d* = 0.018). We also examined the continuous relationship between task performance and MMN amplitude through Spearman’s correlation analysis under each visual demand condition, which similarly yielded no significant association (low demand: ρ = –0.125, *p* = 0.415; high demand: ρ = –0.096, *p* = 0.529). These findings suggest that the MMN differences observed in our study were not simply driven by poorer task performance among children with ADHD.

**Supplement 6. Time-frequency results from cluster-based permutation *t* test**

For the frontal theta ITPC difference, the cluster-based permutation *t test* revealed significant differences from zero for children with ADHD, demonstrating a positive difference under both low- (70–180 ms, cluster *p* < 0.001) and high-demand (0–120 ms, cluster *p* < 0.001) conditions in the visual task (Figure S4A), suggesting greater frontal phase consistency across deviant trials than across standard trials. In contrast, TD children showed a significant negative difference from zero in both conditions (low demand: 0–270 ms; high demand: 0–350 ms, cluster *p* < 0.001), indicating greater phase consistency across standard trials than across deviant trials. A significant positive frontal theta MI was observed in children with ADHD for both low- (120–350 ms, cluster *p* < 0.001) and high-demand (120–400 ms, cluster *p* < 0.001) conditions (Figure S4B), revealing stronger frontal theta power for auditory deviant tones than to standard tones in children with ADHD. However, the theta MI in TD children was not significantly different from zero for either condition (*p*s > 0.05).

**Supplement 7. Permutation-based corrected correlation results**

We applied a permutation test with 1,000 iterations by randomly shuffling symptom scores to assess the correlations between EEG indices and symptom severity ^8–10^. For each permutation, the most extreme absolute correlation coefficient (i.e., the most positive or the most negative ρ among the correlation coefficients) was extracted to form a null distribution. The observed correlation coefficient was considered significant if it exceeded either the top or bottom 2.5% of values in the null distribution, corresponding to a two-tailed family-wise error rate (FWER) corrected α level of 0.05. Notably, after correction for multiple comparisons, the correlation between inattention scores and MMN latency in the low-demand condition remained significant (permutation-based *p* = 0.048), and the correlation between hyperactivity/impulsivity scores and MMN amplitude reached marginal significance (permutation-based *p* = 0.066). All corrected *p* values are provided in Table S2.

**Supplemental** **References**

1. Petersen B, Andersen ASF, Haumann NT, *et al.* The CI MuMuFe – A New MMN Paradigm for Measuring Music Discrimination in Electric Hearing. Front Neurosci. 2020;14. https://doi.org/10.3389/fnins.2020.00002

2. Schröger E. The influence of stimulus intensity and inter-stimulus interval on the detection of pitch and loudness changes. Electroencephalogr Clin Neurophysiol Potentials Sect. 1996;100(6):517-526. https://doi.org/10.1016/S0168-5597(96)95576-8

3. Wang E, Sun L, Sun M, *et al.* Attentional selection and suppression in children with attention-deficit/hyperactivity disorder. Biol Psychiatry Cogn Neurosci Neuroimaging. 2016;1(4):372-380. https://doi.org/10.1016/j.bpsc.2016.01.004

4. Kim M, Kim T, Hwang WJ, *et al.* Forecasting prognostic trajectories with mismatch negativity in early psychosis. Psychol Med. 2023;53(4):1489-1499. https://doi.org/10.1017/S0033291721003068

5. Kool L, Oranje B, Meijs H, *et al.* Event-related potentials and use of psychotropic medication in major psychiatric disorders. Psychiatry Res. 2022;314:114637. https://doi.org/10.1016/j.psychres.2022.114637

6. Mahajan Y, McArthur G. The effect of a movie soundtrack on auditory event-related potentials in children, adolescents, and adults. Clin Neurophysiol. 2011;122(5):934-941. https://doi.org/10.1016/j.clinph.2010.08.014

7. Xu W, Monachino AD, McCormick SA, *et al.* Advancing the reporting of pediatric EEG data: Tools for estimating reliability, effect size, and data quality metrics. Dev Cogn Neurosci. 2024;70:101458. https://doi.org/10.1016/j.dcn.2024.101458

8. Groppe DM, Urbach TP, Kutas M. Mass univariate analysis of event-related brain potentials/fields I: A critical tutorial review. Psychophysiology. 2011;48(12):1711-1725. https://doi.org/10.1111/j.1469-8986.2011.01273.x

9. Lum JAG, Byrne LK, Barhoun P, *et al.* Resting state electroencephalography power correlates with individual differences in implicit sequence learning. Eur J Neurosci. 2023;58(3):2838-2852. https://doi.org/10.1111/ejn.16059

10. Seeber M, Cantonas LM, Hoevels M, Sesia T, Visser-Vandewalle V, Michel CM. Subcortical electrophysiological activity is detectable with high-density EEG source imaging. Nat Commun. 2019;10(1):753. https://doi.org/10.1038/s41467-019-08725-w

**Table S1: Reliability for MMN and P3a Amplitudes and Latencies Across Groups and Conditions**

| ERP measures | ADHD | |  | TD | |
| --- | --- | --- | --- | --- | --- |
|  | Low demand | High demand |  | Low demand | High demand |
| MMN amplitude | 0.755 | 0.813 |  | 0.810 | 0.773 |
| MMN latency | 0.711 | 0.790 |  | 0.748 | 0.770 |
| P3a amplitude | 0.718 | 0.771 |  | 0.831 | 0.786 |
| P3a latency | 0.803 | 0.782 |  | 0.724 | 0.729 |

**Note:** ADHD = attention-deficit/hyperactivity disorder; ERP = event-related potential; MMN = mismatch negativity; TD = typically developing.

**Table S2: Permutation-based Corrected *p* Values for Correlations in Children With ADHD**

| ERP measures | Inattention scores | |  | Hyperactivity/impulsivity scores | |
| --- | --- | --- | --- | --- | --- |
|  | Low demand | High demand |  | Low demand | High demand |
| MMN amplitude | ρ = 0.021,  *p* = 0.940 | ρ = 0.082,  *p* = 0.940 |  | ρ = –0.324,  *p* = 0.066^+^ | ρ = –0.210,  *p* = 0.368 |
| MMN latency | ρ = –0.338,  *p* = 0.048^*^ | ρ = –0.089,  *p* = 0.940 |  | ρ = –0.174,  *p* = 0.518 | ρ = –0.018,  *p* = 0.940 |
| P3a amplitude | ρ = –0.025,  *p* = 0.940 | ρ = –0.011,  *p* = 0.940 |  | ρ = 0.015,  *p* = 0.940 | ρ = –0.034,  *p* = 0.940 |
| P3a latency | ρ = 0.147,  *p* = 0.740 | ρ = –0.111,  *p* = 0.998 |  | ρ = 0.188,  *p* = 0.460 | ρ = 0.034,  *p* = 0.940 |

**Note:** ADHD = attention-deficit/hyperactivity disorder; ERP = event-related potential; MMN = mismatch negativity.

^*^*p* < .05, ^+^*p* < .10, permutation-based.

**
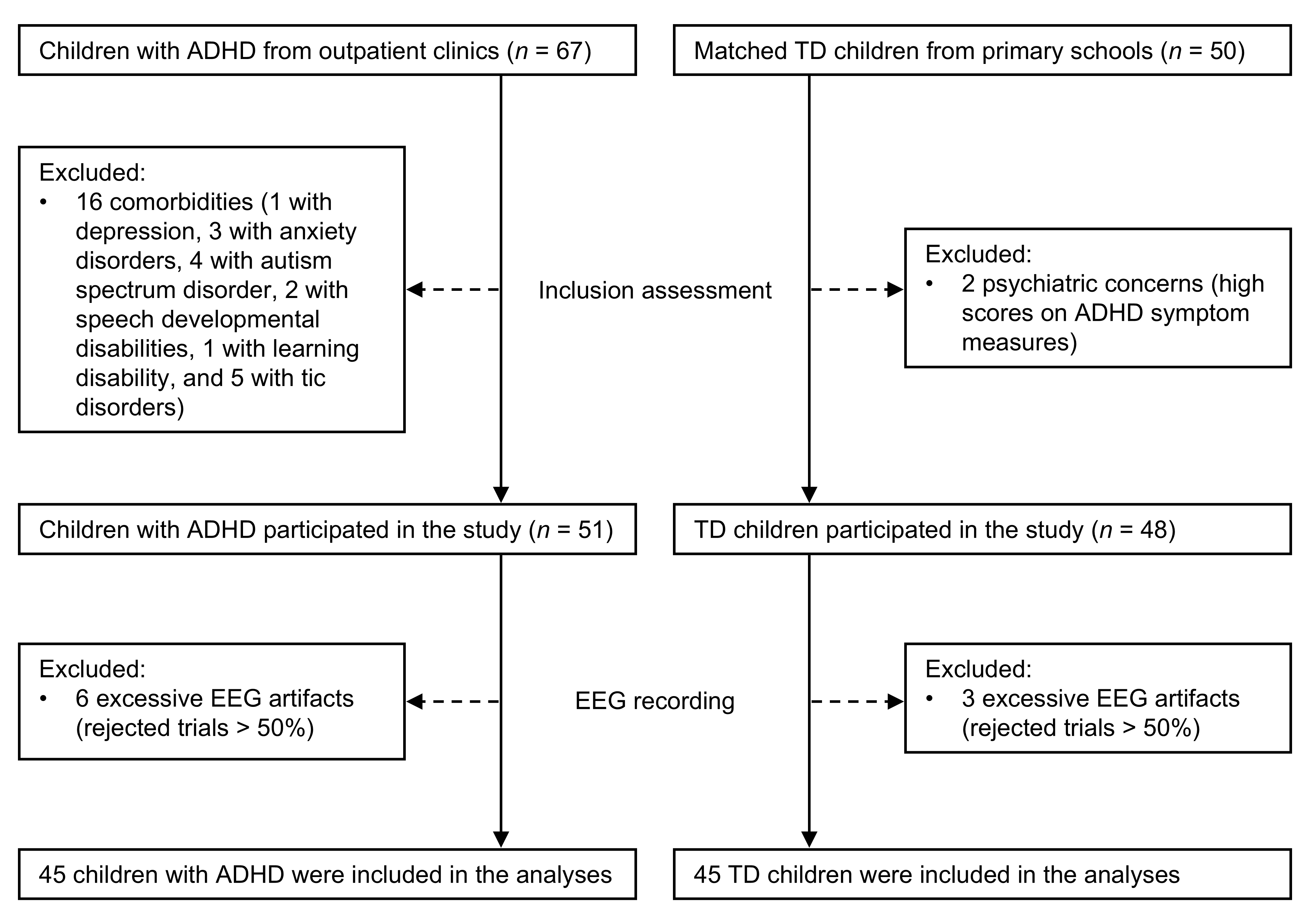
**

**Figure S1: Flowchart of Participant Recruitment and Exclusion**

**Note:** ADHD = attention-deficit/hyperactivity disorder; EEG = electroencephalography; TD = typically developing.

**
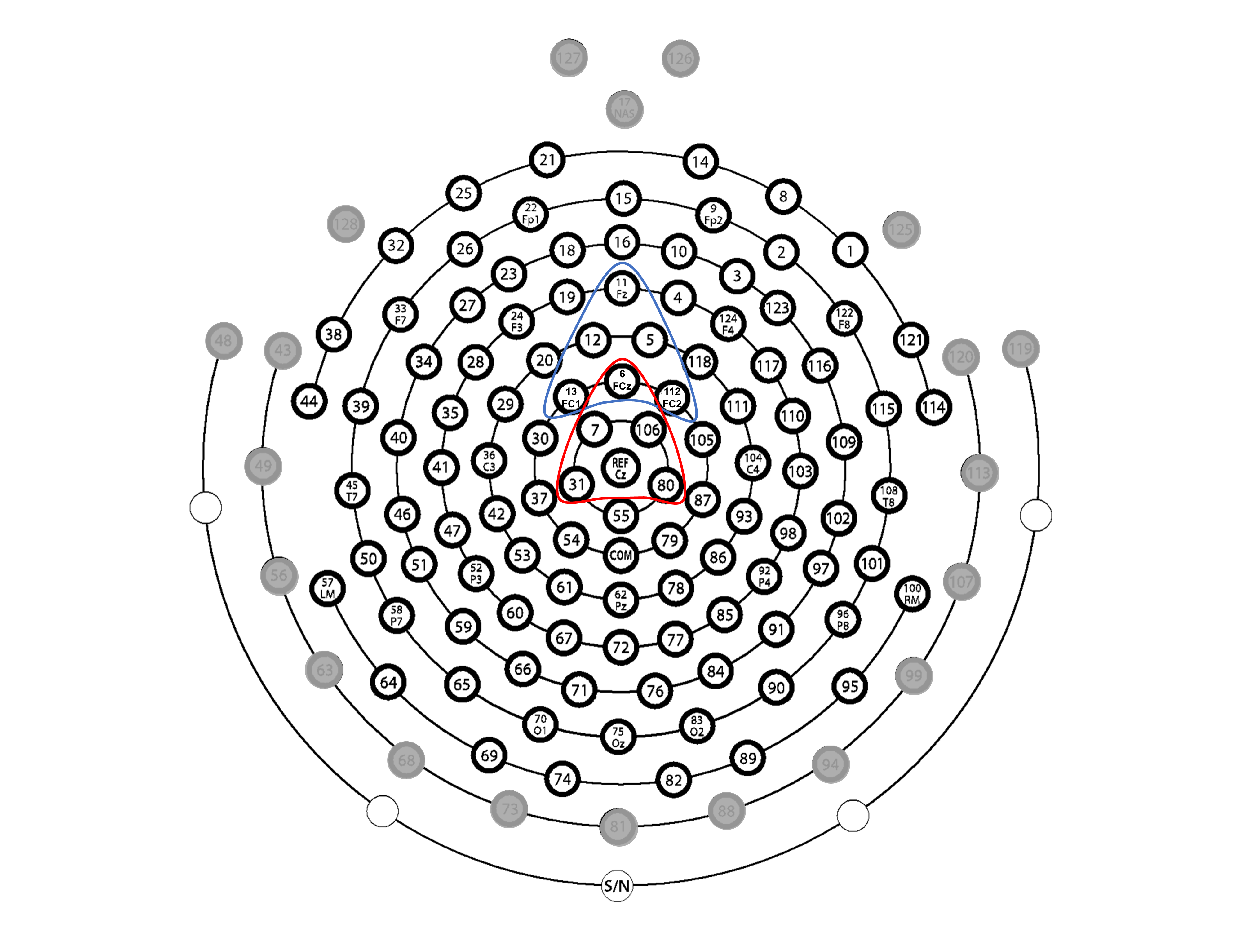
**

**Figure S2: Electrode positions of the EGI 128-channel Geodesic Sensor Net.**

**Note:** The shaded areas indicate the 20 outermost electrodes that were excluded from the analyses. The blue areas show the frontal electrodes that were used for the MMN analysis. The red areas show the central electrodes that were used for the P3a analysis.

**
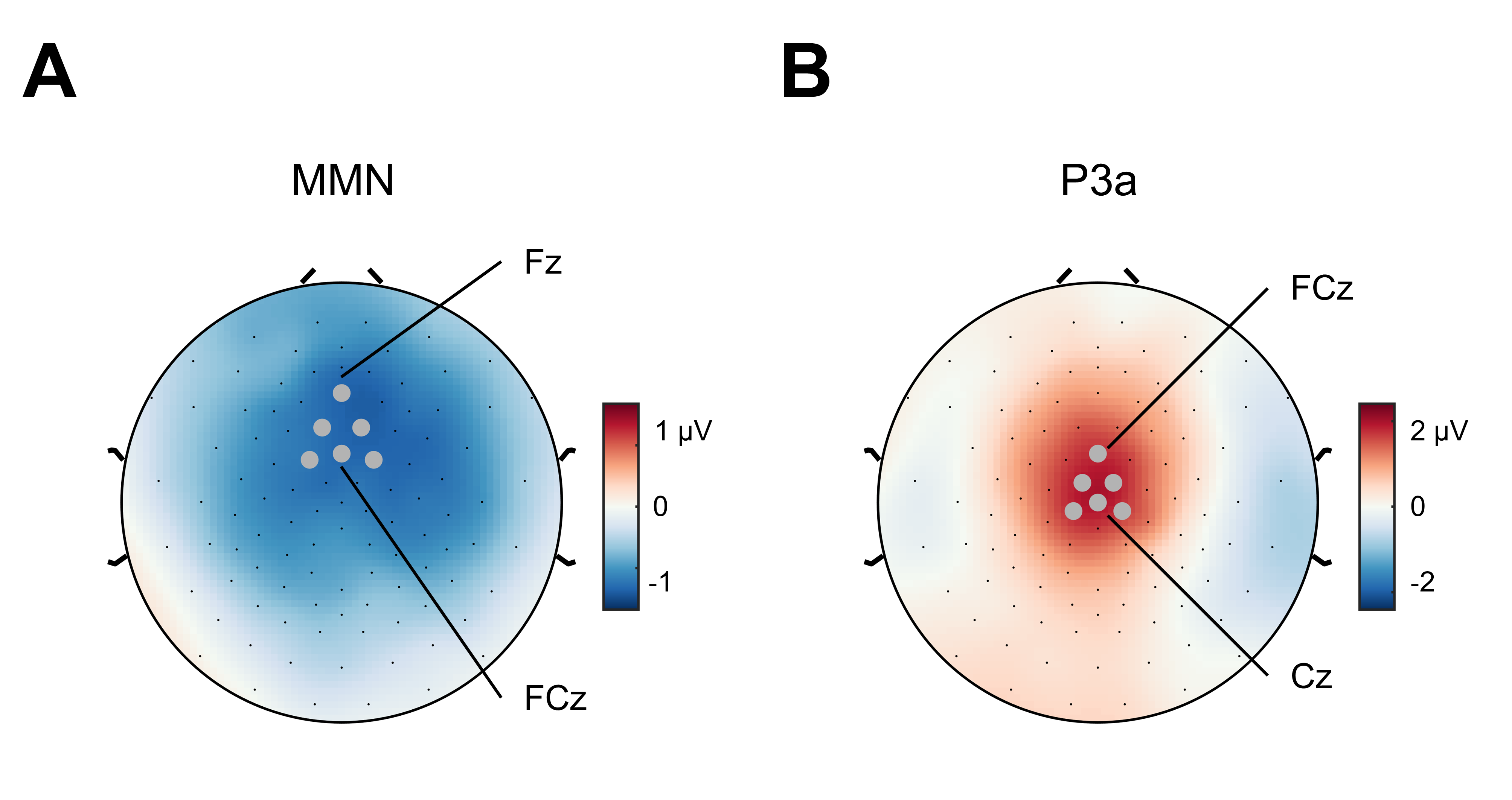
**

**Figure S3: Grand-Grand Average for** **the MMN and P3a Components.**

**Note:** (A) Topographical maps of the MMN component collapsed across all groups and conditions during the 100–250 ms. Grey dots indicate the frontal electrodes (Fz, 5, 12, FC1, FCz, FC2) showing the maximal MMN activity. (B) Topographical maps of the P3a component collapsed across all groups and conditions during the 250–350 ms. Grey dots indicate the central electrodes (FCz, 7, 31, Cz, 80, 106) showing the maximal P3a activity. MMN = mismatch negativity.


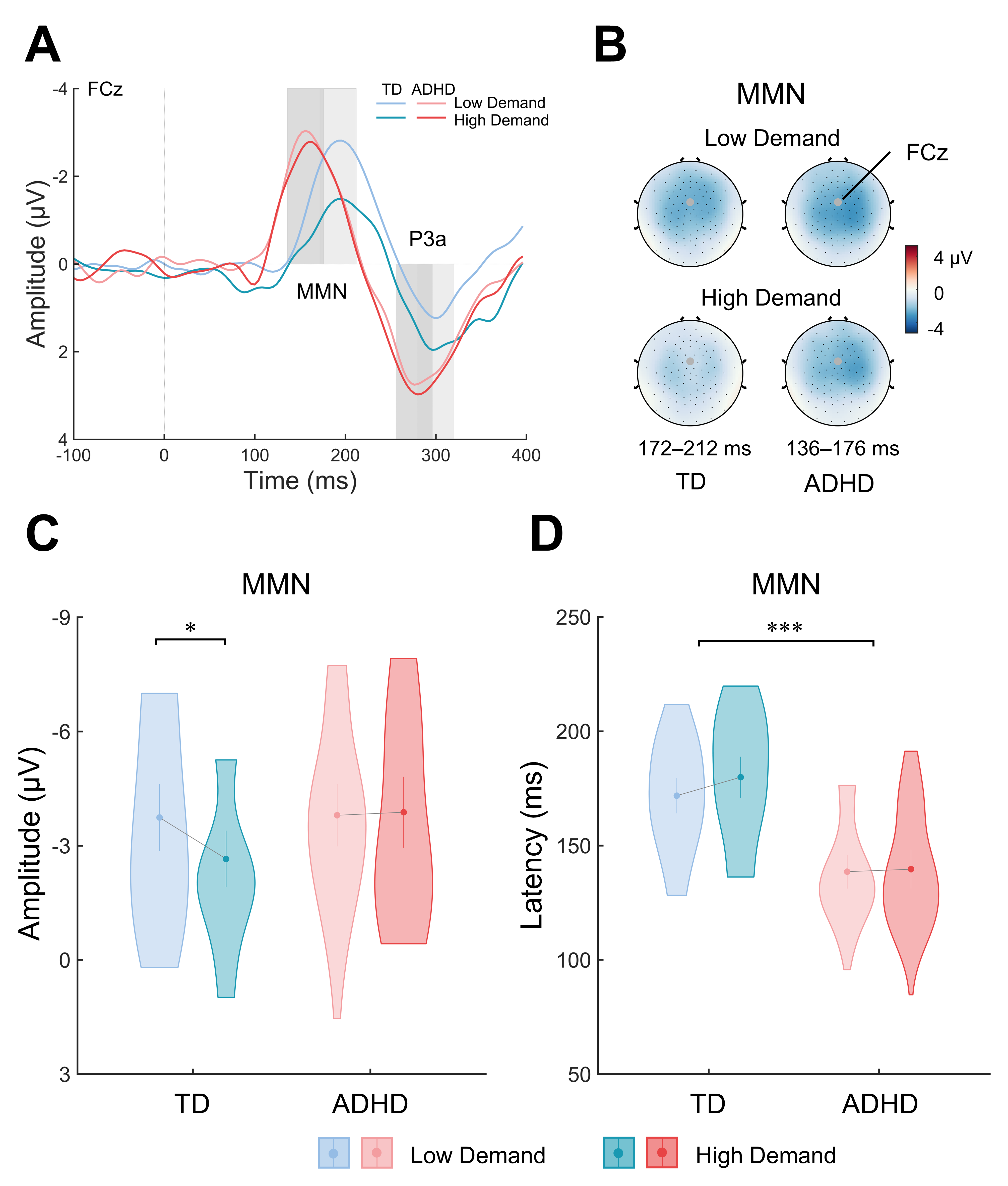


**Figure S4: Results of FCz-Based Analysis for the MMN Component**

**Note:** (A) Grand average difference waves (deviant – standard) at the FCz electrode for the TD and ADHD groups under low- and high-demand conditions. The vertical dotted line at 0 ms indicates the time of stimulus onset. The light and dark gray rectangular areas represent the time windows used to construct topographical maps of the MMN and P3a components for both groups. (B) Topographical maps of the MMN component for the TD and ADHD groups in the low- and high-demand conditions. (C) Amplitudes of the MMN component for the TD and ADHD groups in the low- and high-demand conditions. A significant interaction effect between group and visual demand on MMN amplitude was observed, revealing that TD children presented a smaller amplitude in the high-demand condition, but this effect was not observed in children with ADHD. (D) Latencies of the MMN component for the TD and ADHD groups in the low- and high-demand conditions. Compared with TD children, children with ADHD had significantly earlier MMN latencies. ADHD = attention-deficit/hyperactivity disorder; MMN = mismatch negativity; TD = typically developing.

**p* < .05, ****p* < .001.

**
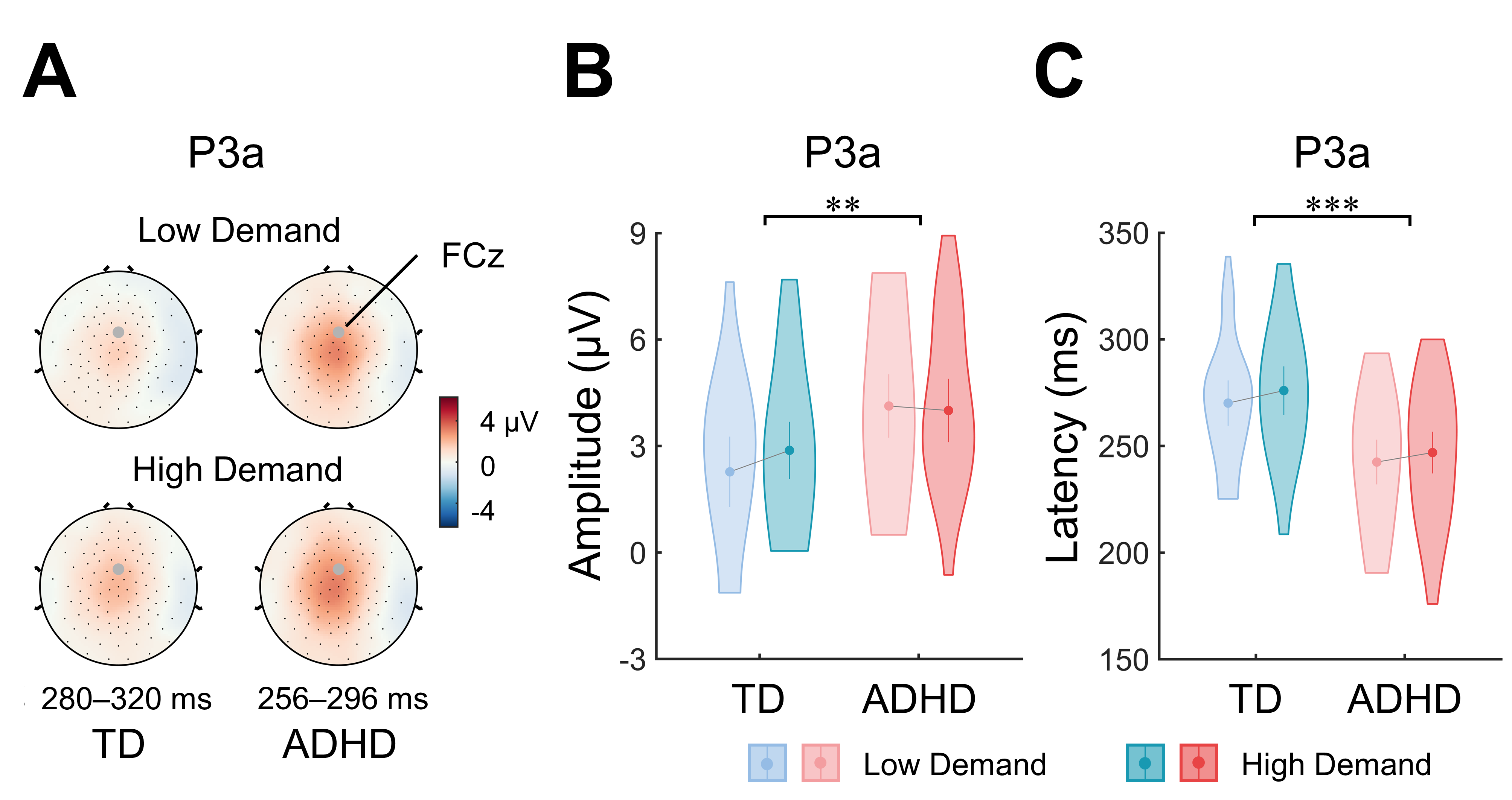
**

**Figure S5: Results of FCz-Based Analysis for the P3a Component**

**Note:** (A) Topographical maps of the P3a component for the TD and ADHD groups in the low- and high-demand conditions. (B) Amplitudes of the P3a component for the TD and ADHD groups in the low- and high-demand conditions. Compared with TD children, children with ADHD presented a greater P3a amplitude. (C) Latencies of the P3a component for the TD and ADHD groups in the low- and high-demand conditions. Compared with TD children, children with ADHD presented earlier P3a latency. ADHD = attention-deficit/hyperactivity disorder; TD = typically developing.

***p* < .01, ****p* < .001.


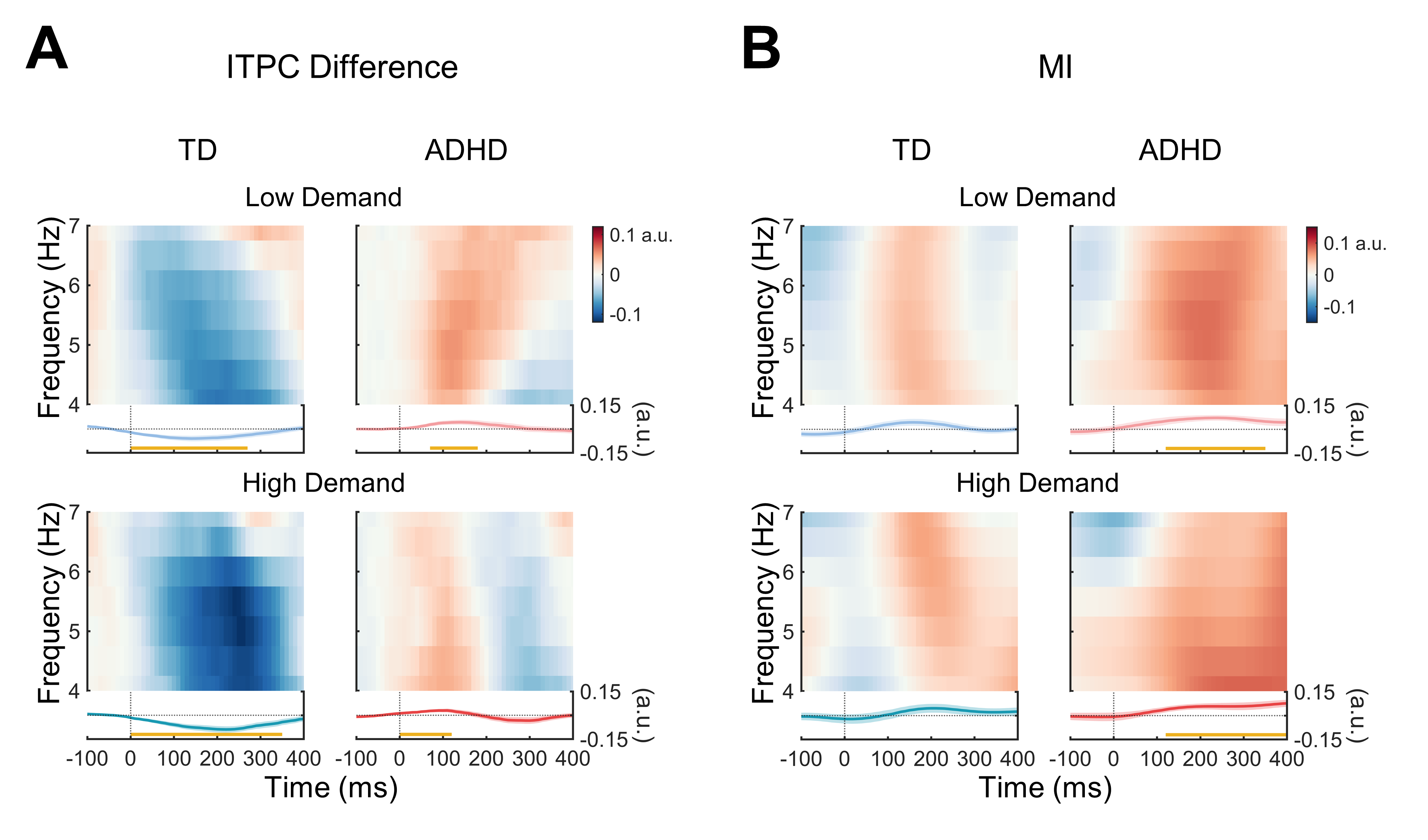


**Figure S6: Time-Frequency Results From Cluster-Based Permutation *t* Test**

**Note:** (A) The time-frequency surfaces of the ITPC difference (deviant – standard) for the TD and ADHD groups in the low- and high-demand conditions, with the time course of the ITPC difference (averaged over 4–7 Hz) displayed below. Positive values indicate greater phase consistency across deviant trials, whereas negative values indicate greater phase consistency across standard trials. The yellow lines at the bottom indicate periods where the theta ITPC difference significantly differed from zero (cluster *p* < .001). (B) The time-frequency surfaces of MI for the TD and ADHD groups in the low- and high-demand conditions, with the time course of the MI (averaged over 4–7 Hz) displayed below. Positive values indicate stronger neural responses to deviant tones. The yellow lines at the bottom indicate periods where the theta MI significantly differed from zero (cluster *p* < .001). Light-colored shading represents error bars (±1 *SE*) in all plots. ADHD = attention-deficit/hyperactivity disorder; ITPC = intertrial phase coherence; MI = modulation index; TD = typically developing.

**
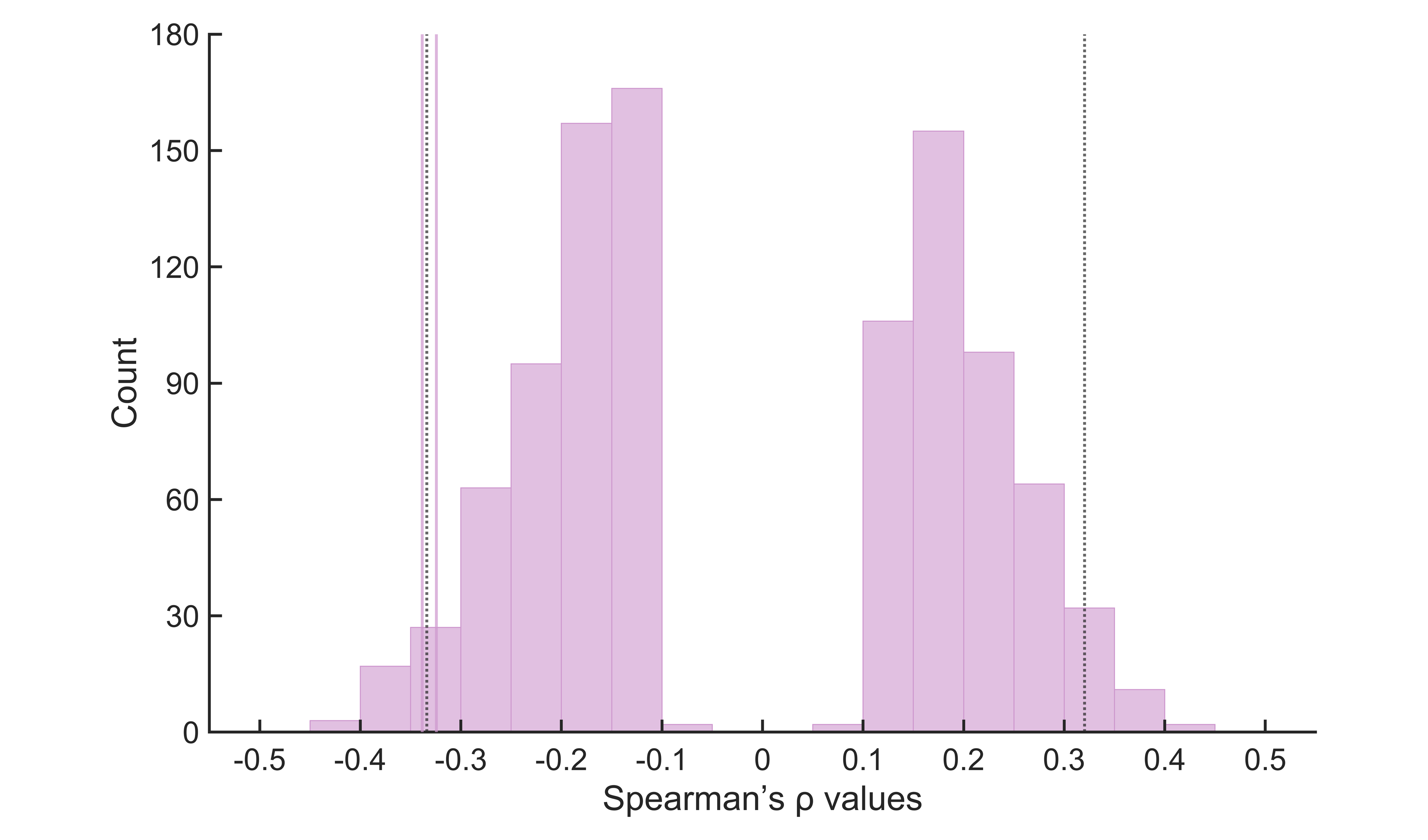
**

**Figure S7: Permutation-Based Corrected Correlation Results in Children With ADHD**

**Note:** Null distributions of Spearman’s ρ values were generated using a permutation test with 1,000 iterations by randomly shuffling symptom scores. Dotted lines indicate the upper and lower 2.5% of the null distribution. Solid lines represent the observed (uncorrected) ρ values for the correlations between hyperactivity/impulsivity scores and MMN amplitude, and between inattention scores and MMN latency in the low-demand condition (permutation-based *p* = 0.066 and 0.048, respectively). ADHD = attention-deficit/hyperactivity disorder; MMN = mismatch negativity.
